# An overview of the sensory receptors regulating cough

**DOI:** 10.1186/1745-9974-1-2

**Published:** 2005-08-04

**Authors:** Stuart B Mazzone

**Affiliations:** 1 Howard Florey Institute University of Melbourne Parkville VIC 3010 Australia

**Keywords:** Cough Receptor, Nociceptor, Rapidly Adapting Receptor, Mechanosensor, Airway, Chemosensor

## Abstract

The cough reflex represents a primary defensive mechanism for airway protection in a variety of mammalian species. However, excessive and inappropriate coughing can emerge as a primary presenting symptom of many airway diseases. Cough disorders are characterized by a reduction in the threshold for reflex initiation and, as a consequence, the occurrence of cough in response to stimuli that are normally innocuous in nature. The current therapeutic strategies for the treatment of cough disorders are only moderately effective. This undoubtedly relates in part to limitations in our understanding of the neural components comprising the cough reflex pathway. The aim of this review is to provide an overview of current concepts relating to the sensory innervation to the mammalian airways, focusing particularly on the sensory receptors that regulate cough. In addition, the review will highlight particular areas and issues relating to cough neurobiology that are creating controversy in the field.

## Introduction

The basic nature of the respiratory system (i.e., inspiration of air from the surrounding environment for gas exchange), as well as the shared nature of the initial anatomical structures for the passage of food and air, places the airways and lungs under the constant threat of exposure to a variety of harmful airborne particles, organisms and other substances as well as aspirated gastric contents or accidental inhalation of foodstuffs. It is therefore not surprising that a variety of defensive mechanisms have evolved along with the normal function of the respiratory system to help protect against such threats. Airway protection relies upon specialized epithelial barriers and immune responses as well as a variety of highly co-ordinated neural reflex responses that help to limit the degree of potential harm and ultimately remove or expel the harmful substance from the airways.

Perhaps the most widely recognized neural response involved in airway protection is coughing. Coughing is generally characterized by a reflex-evoked modification of breathing pattern in response to airway irritation [1]. Reflex cough occurs when subsets of airway afferent (sensory) nerves are activated by inhaled, aspirated or locally produced substances. These afferent nerves provide modifying inputs to the brainstem neural elements controlling respiration, and consequently help generate the cough respiratory pattern [1-3]. Although widely studied for many years, there has been much debate surrounding the identity of the airway afferent nerve subtype that precipitates reflex coughing (see below). In addition, cough can also be initiated voluntarily. Little is known about the cortical pathways responsible for voluntary coughing, although they likely share similarities with those pathways responsible for voluntary breath holding and other conscious modifications of respiration. This review will focus on the current understanding of the anatomical and physiological arrangement of the sensory components responsible for reflex coughing. In addition the review will highlight how modifications of the sensory pathways from the airways could lead to inappropriate coughing in disease.

### Classification of afferent nerve fiber types innervating the airways and lungs

Before describing which afferent nerve fibers are involved in reflex coughing, it seems appropriate to first provide a brief overview of the various afferent nerve subtypes that have been described in the mammalian airways. For the purposes of this review, much of the classification of airway afferents will relate to information gained from studies employing guinea pigs, the most widely utilized species with respect to airway innervation and cough. Whether studies in guinea pigs (or indeed any other experimental animal) can be directly translated to humans is a subject for additional debate. The discussion will also be restricted to only those afferent fibers that innervate the airways caudal to (and including) the larynx.

Airway sensory nerves do not form a homogeneous population. However, to date, there is no single classification scheme that adequately and unambiguously subcategorizes the various afferent nerve subtypes that have been described in the airways. Although a functional classification is commonly employed (describing the physiological responsiveness of airway afferents), subtypes can be alternatively delineated based on their origin, location in the airways, neurochemistry, electrophysiological properties or by the reflexes that are evoked secondary to afferent activation [4]. This lack of a universal classification scheme, coupled with attempts to classify an afferent subtype using only one phenotypic trait, often leads to some confusion as to the identity of a given afferent nerve type. It is therefore desirable to consider multiple characteristics when defining an airway afferent fiber.

**Figure 1:**
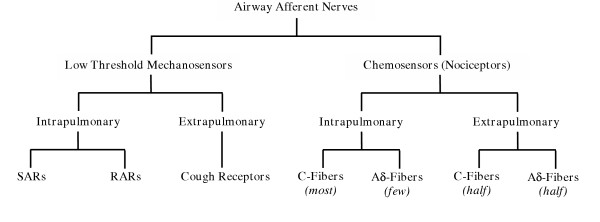
Basic schematic classification of afferent nerve subtypes innervating the guinea pig airways. Abbreviations: RAR; rapidly adapting airway mechanoreceptor; SAR, slowly adapting airway mechanoreceptor.

In guinea pigs (and likely true for all mammals) airway sensory nerves can be broadly functionally classified as either primarily mechanically sensitive (low threshold mechanosensors) or primarily chemically sensitive (chemosensors or alternatively, nociceptors) (Fig 1). Low threshold mechanoreceptors are readily activated by one or more mechanical stimuli, including lung inflation, bronchospasm or light touch, but generally do not respond directly to chemical stimuli unless the stimulus acts upon airway structural cells to result in mechanical distortion of the nerve terminal [5-8]. Conversely, chemosensors are typically activated directly or sensitized by a wide range of chemicals, including capsaicin, bradykinin, adenosine, PGE2, but are relatively insensitive to mechanical stimuli [9,10]. This broad delineation, however, may not be strictly correct as at least some low threshold mechanosensors also directly respond to chemical stimuli, including acid and ATP, although these mediators may still activate the nerve terminal via mechanical mechanisms [11,12]. Subtypes of both the mechanosensors and chemosensors are readily identified (described below). Regardless of the afferent fiber, the majority of airway afferent nerves originate in the vagal sensory ganglia (nodose or jugular) [13,14]. A small population of fibers (believed to be a subpopulation of chemosensitive nerves) may have their origin in dorsal root ganglia adjacent to the upper thoracic spinal cord [15]. Little is known about the role of spinal afferents in airway defense.

#### Low threshold mechanosensors

Two classic types of low threshold mechanosensors have been described in the intrapulmonary airways of a number of mammalian species, namely the rapidly adapting receptors (RARs) and slowly adapting receptors (SARs) [8,9,16-20]. However, when comparing only a limited number of phenotypic traits RARs and SARs may appear indistinguishable (Table 1). Thus, RARs and SARs both originate in the nodose ganglia, terminate in the intrapulmonary airways and lung parenchyma, conduct action potentials in the Aβ-range (10–20 m/s) and are sensitive to many mechanical stimuli, including changes in lung volume, airway smooth muscle constriction and airway wall oedema [9,12,17-21]. Accordingly, RARs and SARs may both display activity when the lungs are inflated [9,16-19]. RARs and SARs are also both generally insensitive to a wide range of chemical stimuli, unless the stimulus evokes coincidental changes in airway smooth muscle tone, mucus secretion or airway wall volume [8,17,19].

**Table 1:** Properties of low threshold mechanosensor subtypes innervating the guinea pig airways.

	**SAR**	**RAR**	**Cough Receptor**
***Anatomical Characteristics:***			
**Ganglionic Origin**	Nodose	Nodose	Nodose
**Extrapulmonary Termination**	No	No	Yes
**Intrapulmonary Termination**	Yes	Yes	Few
**Substance P Expression**	No	No	No
**TRPV1 Expression**	No	No	No
			
***Functional Characteristics:***			
**Conduction Velocity (m/sec)**	~18 (Aβ)	~15 (Aβ)	~5 (Aδ)
**Mechanical Threshold**	Low	Low	Low
**Sensitive to:**			
**Punctate Mechanical**	Yes	Yes	Yes
**Capsaicin**	Yes^1^	Yes^1^	No
**Hypertonic Saline**	Unknown	Unknown	Yes
**Bradykinin**	Yes^1^	Yes^1^	No
**Acid**	No	Unknown	Yes
**Inflation (≤50 cmH_2_O)**	Yes	Yes	No
**Deflation/Collapse**	No	Yes	No
**Stretch**	Yes	Yes	No
**Bronchoconstriction**	Yes	Yes	No
**ATP**	Yes	Yes	No
**Reflex Effects on Respiration**	Hering-Breuer	Tachypnea	Cough

^1 ^SARs and RARs are insensitive to the direct action of these chemicals on the nerve terminal. However, chemical stimuli such as capsaicin and bradykinin can activate SARs and RARs secondary to airway smooth muscle contraction, mucous secretion or edema formation. Cough receptors are insensitive to both the direct and indirect actions of capsaicin and bradykinin. See text for references.

Nevertheless, RARs and SARs can be differentiated by comparing their individual mechanical activation profiles, mechanical adaptation properties, central termination patterns and the reflexes that each precipitate (Table 1). Thus, RARs may be activated during both inflation and deflation of the lungs (including lung collapse) [9,17]. SARs, on the other hand, display activity during tidal inspirations, peaking just prior to the initiation of expiration [9,16]. As their names suggest, RARs display rapid adaptation (i.e., a rapid reduction in the number of action potentials) during sustained lung inflations, whereas SARs adapt slowly to this stimulus [9,17]. It is important to note, however, that this rapid adaptation shown by RARs during sustained lung inflations is unlikely an electrophysiological property of the nerve terminal but rather relates to the nature of the stimulus. RARs typically adapt slowly to other types of mechanical stimuli, including dynamic lung inflations, bronchospasm and lung collapse [12,19]. Finally, activation of RARs evokes tachypnea and airway smooth muscle constriction, whereas SARs are likely the primary afferent fibers involved in the Hering-Breuer reflex, which terminates inspiration and initiates expiration when the lungs are adequately inflated [16,17]. SAR activation also inhibits cholinergic drive to the airway smooth muscle, resulting in a reduction in airway tone [8]. The different reflexes that are evoked by these afferent nerve subtypes likely reflect the distinct brainstem neurons innervated by RARs and SARs [reviewed in 22].

**Figure 2:**
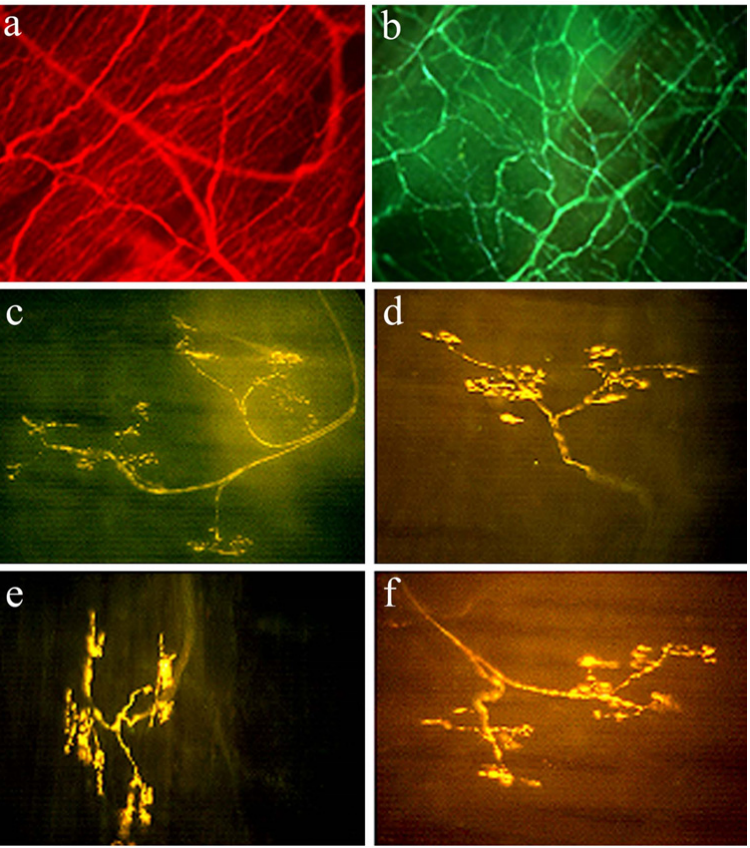
Photomicrographs of the guinea pig trachea showing (a) all nerve fibers immunostained for the pan neuronal marker Protein Gene Product 9.5; (b) jugular ganglia derived chemosensitive C-fiber plexus immunostained for substance P and (c-f) four representative nodose ganglia-derived low threshold mechanosensors (putative cough receptors) stained using the Fluorescent Marker (FM) 2–10. Note the clear distinction between the terminal arrangements of airway C-fibers and cough receptors. The terminal structure of guinea pig SARs, RARs and Aδ-chemosensors is presently unknown. Magnification: X40 (a), X100 (b) and X200 (c-f).

A third type of low threshold mechanosensor has been described in the guinea pig airways [12]. These fibers also originate from the nodose ganglia, but are primary located in the extrapulmonary airways (larynx, trachea and large bronchi) and are quite distinct to RARs and SARs (Figure 2; Table 1). Extrapulmonary low threshold mechanosensors are exquisitely sensitive to punctate mechanical stimuli (such as touch) but are insensitive to physiologically-relevant tissue stretching, changes in luminal pressure or airway smooth muscle constriction [12]. Extrapulmonary low threshold mechanosensors are also readily differentiated from their intrapulmonary counterparts by a much slower conduction velocity (~5 m/sec, Aδ-range) and a lack of sensitivity to the purinergic agonist ATP [12]. During sustained punctate mechanical stimulation, extrapulmonary mechanosensors display rapid adaptation, although again this likely reflects some property of the mechanics of the stimulus in relation to tissue surrounding the nerve terminal rather than reflecting electrophysiological adaptation [23]. Circumstantial evidence suggests that analogous fibers may be present in the extrapulmonary airways of cats, dogs and humans [2,24-30]. It is presently unknown whether this mechanosensor subtype is activated during normal breathing.

#### Chemosensors

Chemically-sensitive airway afferent fibers are found throughout the airways and lungs and are generally quiescent in the normal airways, becoming recruited during airways inflammation or irritation. Airway chemosensors are derived from both the nodose and jugular vagal ganglia, as well as from the dorsal root ganglia [13-15]. As described above, chemosensors are typically defined by the ability of a variety of chemicals to directly activate the nerve terminal (i.e., not secondarily to structural alterations within the tissue; Table 2). However, care needs to be taken when differentiating an airway chemosensor form other airway afferent nerve subtypes. For example, often airway chemosensors are stereotypically defined by their responsiveness to the irritant chemical capsaicin and, hence, the expression of the capsaicin receptor (TRPV1). This definition, however, is not strictly accurate, as at least some species possess capsaicin-insensitive, TRPV1-negative chemosensors [31]. Alternatively, it may be assumed that all airway chemosensors are C-fiber type nociceptors. This is also incorrect, as airway (and other visceral) chemosensors that conduct action potentials in the Aδ-fiber range have been identified (analogous to somatic Aδ-nociceptors) [13,32,33]. Furthermore, due to the overwhelming number of studies conducted in guinea pigs, chemically-sensitive fibers are often presumed to express tachykinins (substance P and/ or neurokinin A) (Fig 2). Guinea pigs are perhaps unique amongst mammals and express a high density of tachykinin-containing airway C-fibers, especially in their extrapulmonary airways [34-36]. Indeed, in the airways of most mammalian species (and in the guinea pig intrapulmonary airways) the majority of C-fiber chemosensors do not express tachykinins [35,36]. Given these reasons, airway chemosensors are sometimes thought of as high threshold mechanosensors. Within this group are fibers that are not readily excited by mechanical stimulation (bronchoconstriction, lung inflations light touch, etc), but can be activated using severe mechanical manipulations (lung hyperinflation, forceful punctate stimuli etc) and one or more chemical stimuli (capsaicin, bradykinin, adenosine etc).

**Table 2:** Properties of chemosensor subtypes innervating the guinea pig airways.

	**C-Fiber**	**C-Fiber**	**Aδ-Fiber**
***Anatomical Characteristics:***			
**Ganglionic Origin**	Nodose	Jugular	Jugular
**Extrapulmonary Termination**	No	Yes	Yes
**Intrapulmonary Termination**	Yes	Yes	Few
**Substance P Expression (%)^1^**	Yes (50)	Yes (90–100)	No (0)
**TRPV1 Expression^2^**	Yes	Yes	Yes
			
***Functional Characteristics:***			
**Conduction Velocity (m/sec)**	<1	<1	~6
**Mechanical Threshold**	High	High	High
**Sensitive to:**			
**Punctate Mechanical**	Yes^3^	Yes^3^	Yes^3^
**Capsaicin**	Yes	Yes	Yes
**Hypertonic Saline**	Unknown	Yes	Yes
**Bradykinin**	Yes	Yes	Yes
**Acid**	Yes	Yes	Yes
**Inflation (≤50 cmH_2_O)**	No	No	No
**Deflation/Collapse**	No	No	No
**Stretch**	No	No	No
**Bronchoconstriction**	No	No	No
**ATP**	Yes	No	No
**Serotonin (5-HT)**	Yes	No	Unknown
**Reflex Effects on Respiration**	Apnea^4^	Apnea^4^	Apnea^4^

^1 ^Percentage of soma expressing substance P shown in parentheses [taken from ref 36]. ^2 ^Functionally responsive to capsaicin and/or TRPV1 detected immunohistochemically. There is no data available indicating percentage of cells expressing TRPV1. ^3 ^All airway afferents are responsive to punctate mechanical stimulation. However, the threshold for activation is approximately 100 fold higher for chemosensors compared to mechanosensors. ^4 ^The basic respiratory reflex evoked by capsaicin is apnea or respiratory slowing, often proceeded by rapid shallow breathing. However, the precise reflex response evoked by each chemosensor subtype has not been described. See text for references.

### Airway afferent nerves and cough

The identity of the afferent nerve fiber subtype that is primarily responsible for evoking reflex coughing has been the subject of much debate. Studies in experimental animals and in humans show clearly that multiple types of mechanical and chemical stimuli can (under the right experimental conditions) evoke coughing [1,12,24-30,37,38]. This would argue that multiple afferent nerve subtypes (mechanosensors and chemosensors) might be involved in the production of reflex coughing. However, not all stimuli evoke cough under all conditions [3,12]. This might suggest divergence between multiple reflex pathways or the existence of primary and secondary cough afferent pathways (discussed below).

#### Rapidly adapting receptors (RARs) and chemosensors

RARs have long been presumed to be the primary afferent nerve fibers that evoke defensive cough in the airways [1,4,5,39]. Indeed, it has been proposed that coughing can be initiated following the activation of RARs by airway smooth muscle constriction, mucous accumulation, mechanical irritation and even capsaicin and bradykinin application (due to the resulting airway obstruction) [1,4,17]. However, several observations argue against the role of classic RARs as the primary cough-provoking afferent fibers. For example, many stimuli that produce robust activation of RARs (e.g. thromboxane, leukotriene C_4 _(LTC_4_), histamine, neurokinins, methacholine) are ineffective or only modestly effective at evoking cough [17,28,40-42]. Moreover, in some coughing species (e.g., guinea pigs) many RARs are spontaneously active throughout the respiratory cycle and yet cough is only induced in response to very specific stimuli [8,12,14,19].

Evidence also supports a role of airway chemosensitive nerve fibers in the cough reflex. For example, stimuli that are known to activate airway chemosensors, such as capsaicin, bradykinin and citric acid, are amongst the most potent tussigenic agents in conscious animals and humans [12,26,38,43,44]. However, capsaicin and bradykinin do not evoke cough in anesthetized animals or humans, even though cough can be evoked in these same animals by mechanically probing the airway mucosa [12,25,27]. In fact, in anesthetized animals acute capsaicin challenge has been shown to inhibit breathing and, as a consequence, inhibit cough evoked by mechanical stimulation of the airways [12,25,27]. These conflicting observations have lead to suggestions that in conscious animals cough-evoked by chemosensor stimuli relies on cortical processing of the stimulus, in which the activation of a subset of airway chemosensors generate the conscious perception of airway irritation and promote the urge to cough [3]. Indeed, it is interesting that capsaicin-evoked cough can be consciously suppressed in human subjects [45]. If this hypothesis is correct, then chemosensor-mediated cough may not strictly be reflexive in nature. Rather, the perception of airway irritation may induce the conscious/ voluntary decision to cough. The true respiratory reflex response that is evoked by airway chemosensor stimulation may in fact be rapid inhibition of respiratory activity, which is observed during anesthesia and perhaps over-ridden (unless the reflex is robustly activated) by voluntary control in the conscious state.

#### The guinea pig 'cough receptor': Conflicts and opinions

The recent characterization of an extrapulmonary low threshold mechanosensor in the guinea pig airways (distinct to classic intrapulmonary RARs and SARs) may provide some important insights into the identity of the primary cough-provoking afferent nerve fiber. As described above, these fibers are found within the wall of the larynx, trachea and mainstem bronchi and are functionally differentiated from RARs and SARs by their sensitivity to light punctuate mechanical stimulation, but not to tissue stretch, bronchospasm, ATP and positive/ negative luminal pressures within the physiological range [12]. In addition to touch-like sensitivity, extrapulmonary mechanosensors are also activated by rapid changes in pH (e.g., as might be expected to occur following aspiration of gastric contents) [11,12]. Mechanical irritation and changes in pH are both stimuli that readily evoke cough in conscious and anesthetized animals and humans [11-13,26,46]. This sensitivity profile, their apparent ideal location for airway defense (i.e., in the large airways), the absence of this fiber subtype in species that do not cough (e.g. rats and mice) and several other anatomical and functional observations makes these extrapulmonary low threshold mechanosensors a likely candidate for the primary afferent nerve subtype that evokes reflex defensive coughing in guinea pigs. Accordingly, the term 'cough receptor' has been reintroduced to describe this guinea pig afferent nerve fiber subtype [3,12,47-49].

The identification of a unique afferent fiber subtype involved in generating cough from the guinea pig airways has generated much discussion within the field of cough research. For example, although these extrapulmonary fibers are easily distinguished from classic RARs and SARs in guinea pigs, it is unclear whether analogous fibers exist in the large airways of other species. The observation that the cough reflex can be readily evoked by light touch of the larynx, trachea or mainstem bronchi but not by bronchoconstricting agents, in dogs, cats and humans, provides circumstantial evidence that similar fibers may exist in these species [2,24-30]. It is also presently not known whether cough is the only reflex event initiated by this fiber type, nor is it certain that other fiber types can not produce coughing under some circumstances. However, this fiber type is the only sensory nerve in the guinea pig airways that once activated can initiate cough in both conscious and anesthetized animals [12]. Nevertheless, careful experimentation is required to adequately address these issues.

The appropriateness of employing the term 'cough receptor' to describe the guinea pig extrapulmonary low threshold mechanosensor has also been questioned. Although physiologists commonly describe sensory nerve fiber types as 'receptors' (e.g., muscle stretch receptors, tension receptors, RARs and SARs etc), the term 'receptor' can equally be applied to describe a pharmacological entity (e.g. a G-protein coupled receptor or a ligand-gated ion channel). Given the latter definition, and the observation that capsaicin is one of the most tussigenic stimuli available in conscious animals and humans, it is not surprising that TRPV1 (i.e., the capsaicin receptor) has been identified as a possible 'cough receptor' in guinea pigs and humans [50]. With this approach, any protein responsible for the transduction of a mechanical or chemical stimulus into electrical activity in the sensory nerve terminal (leading to cough) is a pharmacological cough receptor, and therefore a given sensory nerve is likely to have many different cough receptors. However, by defining a protein as a cough receptor it implies that this protein is therefore involved in the cough reflex irrespective of the cell type, tissue or species in which it is expressed. Using the example of TRPV1, it is unlikely that all TRPV1-expressing cells in the airways, and (perhaps with the exception of some nasal and esophageal afferent neurons) improbable that any TRPV1-expressing cells in other tissues or organs are involved in the cough reflex. Furthermore, species such as rats and mice lack the cough reflex, despite possessing numerous TRPV1-positive and capsaicin-sensitive airway afferent nerves [31,51,52].

Although these issues may seem an argument of semantics, they highlight the problems associated with the lack of any standard and widely accepted nomenclature system for defining terms and concepts employed by the field. Given that a 'cough receptor' was defined in the first instance as a putative afferent nerve subtype that evokes cough (and was not obviously intended to be employed to describe a pharmacological entity) [49], it therefore seems appropriate to define the guinea pig extrapulmonary low threshold mechanosensitive afferent nerve subtype as the only cough receptor identified to date.

### Multiple and interacting cough reflex pathways

The breath-to-breath activity of intrapulmonary SARs and RARs is known to play an important role in regulating the excitability of brainstem breathing circuits [53-57]. In addition, activation of bronchopulmonary chemosensors can have profound influences on breathing pattern [12,25,27,58,59]. Given that many of the brainstem neural elements involved in breathing and coughing are shared, it seems therefore logical that alterations in the activity of most airway afferent nerves will play a role in shaping the cough motor pattern, perhaps contributing to different types of cough. For example, the basic primary defensive cough pathway (i.e., uncontrollable cough in response to an acute stimulus such as aspiration or direct mechanical probing of the airway mucosa) is likely mediated primarily by extrapulmonary low threshold mechanosensors (cough receptors). This pathway may therefore represent the primary basic defensive cough reflex pathway that serves to protect the airways from acute assaults. However, cough associated with airways obstruction or more chronic airway irritation (as would be expected to occur in airways disease) may involve the recruitment of other afferent (RAR and/ or chemosensor) pathways. In this scenario, secondary airway afferent pathways may evoke or modify cough responses via interactions with central elements of the primary cough pathway.

One of the problems faced when attempting to study central afferent interactions involved in coughing is the large gap in our understanding of airway sensory nerve integration in the brainstem. Nevertheless, recent studies in guinea pigs have provided some evidence to support the hypothesis that central interactions between cough receptor afferents and airway chemosensors may play an important role in cough reflex hypersensitivity in disease [3,60,61]. In anesthetized guinea pigs, chemosensors play a permissive (but not essential) role in cough evoked by some mechanoreceptor stimulants [62]. Furthermore, activation of normally quiescent airway chemosensors (using capsaicin or bradykinin) does not evoke cough but rather potentiates cough evoked by tracheal cough receptor stimulation [60]. Chemosensor-evoked potentiation of cough may reflect convergence of cough receptors and chemosensors onto common brainstem neurons responsible for generating cough [14,22], and shares many similarities with the interaction between cutaneous mechanosensors and chemosensors in the spinal cord, which is thought to underlie the manifestation of aberrant pain states [Reviewed in 22, 63]. Studies in guinea pigs and humans also suggest that chemosensitive afferent input from the nose or esophagus may heighten cough sensitivity via central interacting mechanisms [64-66].

Combined, these observations suggest that the recruitment of airway or other visceral chemosensors, and the subsequent increase in central cough pathway excitability, may contribute to the hypertussive states that accompany inflammatory diseases of the airways, nose and/ or esophagus. These data also indicate that it may be possible to design future therapeutic strategies that reduce the excitability of secondary cough afferent pathways, thereby treating cough hypersensitivity associated with disease without inhibiting the basic (primary) defensive cough reflex which is essential for airway protection and normal airway functioning.

## Conclusion

Coughing, although essential for protecting the airways from the possible deleterious effects of acute airway irritation, can become excessive and non-productive in many airways diseases. The recent increased interest in cough reflex sensory neurobiology has unveiled a previously unrecognized complexity in the interacting roles of multiple afferent nerve subtypes in regulating this defensive reflex. However, further careful dissection of the cough sensory pathways is still required for the identification of future therapeutic targets for the effective treatment of cough disorders.
